# Visceral Leishmaniasis-Malaria Coinfection and Their Associated Factors in Patients Attending Metema Hospital, Northwest Ethiopia: Suggestion for Integrated Vector Management

**DOI:** 10.1155/2017/6816913

**Published:** 2017-08-28

**Authors:** Getachew Ferede, Ermias Diro, Sisay Getie, Gebeyaw Getnet, Yegnasew Takele, Anteneh Amsalu, Yitayih Wondimeneh

**Affiliations:** ^1^College of Medicine and Health Sciences, School of Biomedical and Laboratory Sciences, Department of Medical Parasitology, University of Gondar, P.O. Box 196, Gondar, Ethiopia; ^2^College of Medicine and Health Sciences, School of Medicine, Department of Internal Medicine, University of Gondar, P.O. Box 196, Gondar, Ethiopia; ^3^Leishmaniasis Research and Treatment Center, University of Gondar, Gondar, Ethiopia; ^4^College of Medicine and Health Sciences, School of Biomedical and Laboratory Sciences, Department of Medical Microbiology, University of Gondar, P.O. Box 196, Gondar, Ethiopia

## Abstract

**Background:**

Despite high prevalence of visceral leishmaniasis and malaria in the study area, their coinfection remains unknown. Therefore, this study was aimed to document VL-malaria coinfections and their associated factors.

**Methods:**

A cross-sectional study was conducted among clinical suspected VL patients attending Metema hospital, Northwest Ethiopia, from January 2014 to June 2014. Blood sample was tested by rk39 antigen-based DiaMed IT-Leish dipstick and Giemsa stain microscopic examination of thick and thin blood smears for malaria detection was performed.

**Result:**

A total of 384 VL suspected patients were included in the study. Out of these, the prevalence of VL was 83 (21.6%) while the prevalence of malaria was 45 (11.7%). Of malaria cases, 40 (89%) were positive for* P. falciparum* and 5 (11%) positive for* P. vivax*. The overall prevalence of VL-malaria coinfection was 16 (4.2%). One-hundred eighty (46.9%) study participants have history of travel. Of these, 10 (5.6%) have VL-malaria coinfections. Age less than 5 years was associated with VL-malaria coinfection.

**Conclusion:**

This study highlights the importance of performing malaria screening amongst VL patients living in malaria-endemic areas, particularly in patients under five years.

## 1. Background

Leishmaniasis and malaria are among the most important six diseases on the World Health Organization (WHO) or Tropical Disease Research list. There are 2 million new cases of Leishmaniasis diagnosed every year [1]. As for malaria, 300–660 million people become infected yearly with the malignant* Plasmodium falciparum*, and 200–300 children are dying every hour from this disease [2, 3]. According to the world malaria report 2016, there were about 212 million cases of malaria and 429,000 deaths [4].

Visceral leishmaniasis (VL) is the phlebotomine sand flies borne disease caused by an intracellular protozoan parasite of the* Leishmania donovani* complex [5], while malaria is* Anopheles* mosquito borne disease caused by an intracellular protozoan parasite of* Plasmodium* species [6]. Usually, the transmission of both parasites occurs when the female insect takes a blood meal. Measures to combat these two diseases usually aimed at interrupting the life-cycle of the parasite by destroying the parasite or its vector. Destroying the parasite could be achieved by vaccination or treatment. Destroying the vector could be achieved by many ways such as introducing an enemy of the vector to the environment, using the sterile male technique, destroying the habitat of the vector, or spraying pesticides [7].

Visceral leishmaniasis and malaria are the two major life-threatening parasitic diseases that still remain a serious public health problem, particularly in endemic areas [8, 9]. Differential diagnosis of VL often includes malaria among other febrile splenomegalies, due to its clinical overlap. Malaria, in fact, is widespread in tropical and subtropical regions of the world, where it accounts for more than 250 million cases annually, the vast majority of which occurs among children under 5 years old [10]. Transmission can occur throughout the year or be seasonal, depending on the region [11]. In the latter case, transmission seasons for VL and malaria may not coincide, but the two diseases still overlap, due to the longer incubation period of VL. The overlap in disease distribution suggests the two diseases could cooccur in the same host.

Visceral leishmaniasis and malaria coinfections have been reported across various African and Asian countries, with the prevalence among VL patients ranging from 20.8% and 6.4% in Uganda [12, 13] to 10.7% in Sudan [14] and 1.2% in Bangladesh [15] and a rate of 5.9% among Indian patients with fever and splenomegaly [16], with the exception of the case-reports [17–20], whose evidence remains anecdotal. Despite high prevalence of the VL [21] and malaria [22] in the study area, their coinfection is unknown. Therefore, this study was designed to document the prevalence of VL-malaria coinfection and their associated factors among patients attending Metema hospital.

## 2. Materials and Methods

### 2.1. Study Area and Period

This study was conducted at Metema hospital from January 2014 to June 2014. Metema is a town in Northwestern Ethiopia, on the border with Sudan. This town is located in the North Gondar Administrative Zone, Amhara region, 897 km North of Addis Ababa and 197 km from the ancient city of Gondar, and it has a latitude and longitude of 12°58′N 36°12′E with an elevation of 685 meters above sea level. This area is malarious and has cases of VL.

### 2.2. Study Design, Study Population, and Sampling Technique

A cross-sectional study was carried out at Metema hospital to determine VL-malaria coinfection among all clinically suspected VL patients. Visceral leishmaniasis suspected patients who have taken treatment for malaria and/or VL for the last two weeks were excluded from the study. Visceral leishmaniasis suspects are defined as those individuals who develop clinical evidence of infection usually have fever, loss of appetite (anorexia), weight loss, fatigue, anemia, enlargement (swelling) of the spleen and liver, and abnormal blood tests [23].

### 2.3. Data Collection and Processing

Well-structured questionnaire was prepared in English version by the research team. It was translated later into the local language, Amharic. The questionnaire addresses patients' sociodemographic information and associated factors. A pretest was conducted among five percent of the total sample size by trained data collectors and any ambiguous questions and repetitive ideas were corrected. Additional response categories were added based on the pretest findings.

### 2.4. Parasitological Techniques

After interviewing the patients, fresh peripheral whole blood sample was tested by the rk39 ICT with sensitivity of 100% and specificity of 98% [24] for diagnosis of VL, and Giemsa stain microscopic examination of thick and thin blood smears for malaria detection and species differentiation was performed systematically.

### 2.5. Data Analysis

Data were entered and analyzed using SPSS version 20 software. The Pearson Chi-square test was employed to examine associated factors with the coinfection. *P* value < 0.05 was considered statistically significant.

### 2.6. Ethical Consideration

Ethical clearance was obtained from Institutional Review Board of University of Gondar. Written informed consent was obtained from each of the volunteer study subjects or guardian of children. Patient information was anonymized and deidentified prior to analysis. Positive results were given for physicians working in the hospital for treatment according to the national treatment guideline.

## 3. Results

### 3.1. Sociodemographic Characteristics

A total of 384 VL suspected participants were included in this study. Out of these, 334 (87%) were males. The mean (standard deviation) age was 28.1 (11.8) years (ranging from 2 to 78 years). Majority 227 (59.1%) of the study participants were in the age group of 15–29 years old. Among the study groups, 230 (59.9%) were illiterate and 267 (69.5%) were farmers followed by 54 (14.1%) daily laborers. The majority of study participants 234 (60.9%) were from rural residents (Table 1).

### 3.2. Visceral Leishmaniasis-Malaria Coinfection and Sociodemographic Characteristics

Out of the total VL suspected cases 2 (33.3%) VL-malaria coinfections were found in the age groups of <5 years followed by 9 (4%) within 15–29 years old (*P* = 0.004). Visceral leishmaniasis-malaria coinfection by gender showed that 15 (4.5%) of males were coinfected by VL-malaria. Individuals who live in the urban 8 (5.3%) and rural 8 (3.4%) were coinfected with VL-malaria. 5 daily laborers (9.3%) were coinfected with VL-malaria (Table 2).

### 3.3. Prevalence of VL-Malaria Coinfection

**Figure 1:**
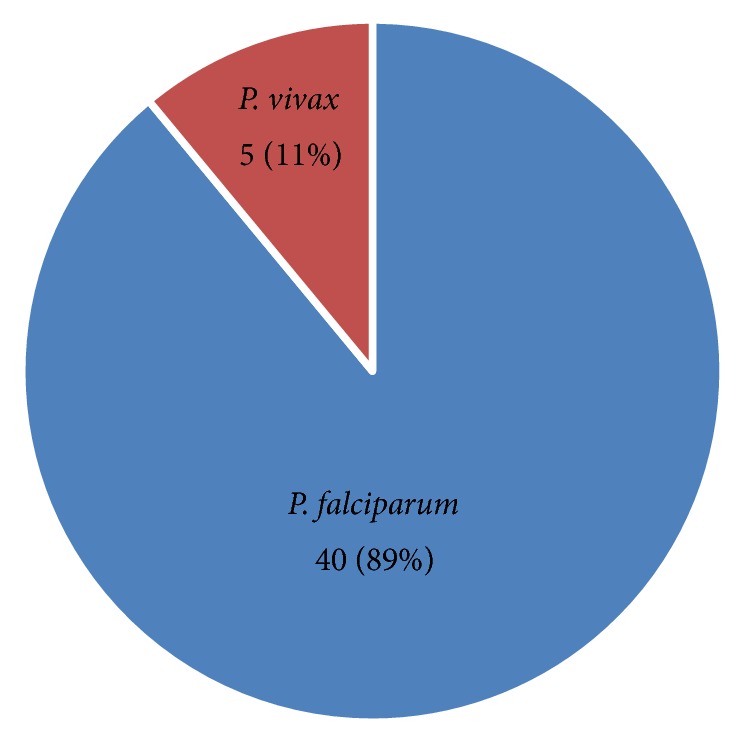
Frequency of* Plasmodium* species at Metema hospital, Northwest Ethiopia, 2014.

Out of the total VL suspected patients, the overall prevalence of VL-malaria coinfection was 16 (4.2%). The prevalence of VL was 83 (21.6%) while the prevalence of malaria was 45 (11.7%) (Table 3). Of the malaria positive patients, 40 (89%) were positive for* P. falciparum* and 5 (11%) were positive for* P. vivax* (Figure 1).

### 3.4. Associated Factors for VL-Malaria Coinfection

From the total study participants, 292 (76%) were used bed nets. Of these, 13 (4.5%) patients were VL-malaria coinfected. Most of the study participants, 217 (56.5%) sleep inside home. One hundred eighty (46.9%) study participants have history of travel from Metema to any region of Ethiopia and vice versa. Of these, 10 (5.6%) of them have VL-malaria coinfection. All study variables did not show statistical significance (Table 4).

## 4. Discussion

Visceral leishmaniasis and malaria coinfection may exist in endemic areas due to similarity in clinical manifestations of the two diseases but their coinfection has been poorly investigated. In the present study, the overall prevalence of VL-malaria coinfection was 4.2%. This is in agreement with the study which is conducted in India 5.9% among Indian patients with fever and splenomegaly [16]. However, this prevalence was lower as compared to study reported in Uganda 19% [25]. This variation might be due to difference in the VL and malaria prevalence which is 21.6% and 11.7% in this study and 57% and 31% in Uganda, respectively [25].

In this study, prevalence of VL-malaria coinfection was nonsignificantly higher in males than females. A similar finding was also reported in other studies [25–27]. The higher prevalence rate might be due to the fact that males engage in activities which make them more prone to infective mosquito and sand fly bites as compared to females who are mostly at home and protected from such infective bites.

Visceral leishmaniasis has been a major health problem in North Gondar, especially in lowlands of Metema and Humera plains, which are important endemic foci in Northwest of Ethiopia [28]. In the present study, its prevalence was 21.6%, which was consistent with the other study done in the same study area, 22.6% [21], however, lower than the study in other areas of Ethiopia which was reported as 39.1% [29]. The variation might be due to heterogeneity of the vectors. Following agricultural development in the region, a large number of labor migrants from the different areas were moved to the endemic areas for crop harvesting. This led to outbreaks of VL, in many areas in the country which resulted in high morbidity and mortality [30].

Malaria is also one of the most public health problems in Ethiopia. The total land mass of the country is regarded as malarious and about 68% of the total population is at risk of malaria infection [31]. In this study, the prevalence of malaria was 11.7%, which is relatively lower than previous study done in the same study area (17%) [22]. The variation might be due to difference in study participants. The predominant* Plasmodium* species detected was* P. falciparum*. This was in agreement with other previous studies [22, 32, 33].

Visceral leishmaniasis-malaria coinfection was significantly higher in patients with the age groups of less than five years (*P* = 0.004) followed by patients in the age groups of fifteen to twenty-nine years. This indicates that children less than five years old are prone to acquire VL-malaria coinfections which might be associated with their low immune system. On the other hand, high prevalence of VL-malaria coinfection within fifteen to twenty-nine years old might be associated with their daily activities. Farming is extensive in Metema due to the fact that young daily laborers move to Metema from different areas for application of herbicide and for gathering of crops. Because of high temperature in this area, daily activities are accomplished especially during night. This may expose them to the bite of mosquitoes and sand fly which means a more concentrated effort should be given to educate these age groups about this disease and its vector.

In this study, majority of study subjects use their bed nets. However, the prevalence of VL-malaria coinfection was high in these subjects. This might be due to improper and infrequent use of their bed net or it might be due to lack of impregnating their bed nets regularly. In the study area most of study participants who were coinfected with VL-malaria were daily laborers followed by merchants. This might be associated with their daily activities which may expose them for vector bite.

This study is limited to the data obtained from the VL suspected patients, which may reduce the prevalence of malaria. Moreover, this study for VL diagnosis depends only on serological tests. There is no microscopically confirmed parasitological data for VL. To fill all these gaps, there is a need for further study including all febrile patients with different study design.

In conclusion, this study tries to see VL-malaria coinfection for the first time and found 4.2% coinfection rate. A significantly higher number of VL-malaria coinfections were showed in study subjects whose age was less than five years old. Based on these findings, we recommend that malaria screening be implemented for all VL patients who live in malaria-endemic areas in order to promptly initiate antimalarial drug treatment. Moreover, to minimize the disease burden, health planners and administrators need to give intensive health education to increase the community awareness about the two diseases' prevention and control strategies.

## Figures and Tables

**Table 1:** Sociodemographic characteristics of the study participants at Metema hospital, Northwest Ethiopia, 2014.

Characteristic		Frequency	Percentage
Age (years)	<5	6	1.6
	5–14	20	5.2
	15–29	227	59.1
	30–44	99	25.8
	≥45	32	8.3

Gender	Male	334	87.0
	Female	50	13.0

Residence	Rural	234	60.9
	Urban	150	39.1

Occupational status	Farmer	267	69.5
	Merchant	12	3.1
	Driver	2	0.5
	Civil servant	12	3.1
	Daily laborer	54	14.1
	Housewife	18	4.7
	Student	19	4.9

Educational status	Illiterate	230	59.9
	Only read and write	85	22.1
	Elementary school	45	11.7
	High school	21	5.5
	College/universities	3	0.8

**Table 2:** Sociodemographic characteristics of the study participants in relation to VL-malaria coinfection at Metema Hospital, Northwest Ethiopia, 2014.

Variables		VL suspected cases		*P* value
		Not VL-malaria coinfected (%)	VL-malaria coinfected (%)	
Age	<5	4 (66.7)	2 (33.3)	0.004
	5–14	20 (100)	0 (0)	
	15–29	218 (96.0)	9 (4.0)	
	30–44	94 (95.0)	5 (5.0)	
	≥45	32 (100)	0 (0)	

Gender	Male	319 (95.5)	15 (4.5)	0.411
	Female	49 (98.0)	1 (2.0)	

Residence	Rural	226 (96.6)	8 (3.4)	0.360
	Urban	142 (94.7)	8 (5.3)	

Occupation status	Farmer	257 (96.3)	10 (3.7)	0.384
	Merchant	11 (91.7)	1 (8.3)	
	Driver	2 (100)	0 (0)	
	Civil servant	12 (100)	0 (0)	
	Daily laborer	49 (90.7)	5 (9.3)	
	Housewife	18 (100)	0 (0)	
	Student	19 (100)	0 (0)	

Educational status	Illiterate	219 (95.2)	11 (4.8)	0.181
	Only read and write	85 (100)	0 (0)	
	Elementary school	42 (93.3)	3 (6.7)	
	High school	19 (90.5)	2 (9.5)	
	Higher education	3 (100)	0 (0)	

**Table 3:** Prevalence of VL, malaria, and their coinfection among VL suspected patients at Metema hospital, Northwest Ethiopia, 2014.

Variables and result	VL (%)	Malaria (%)	VL-malaria coinfection (%)
Negative	301 (78.4)	339 (88.3)	368 (95.8)
Positive	83 (21.6)	45 (11.7)	16 (4.2)

**Table 4:** Associated factors of VL-malaria coinfection at Metema hospital, Northwest Ethiopia, 2014.

Variables	VL suspected cases		*P* value
	Not VL-malaria coinfected (%)	VL-malaria coinfected (%)	
*Bed net use*			
Yes	279 (95.5)	13 (4.5)	0.618
No	89 (96.7)	3 (3.3)	

*Sleep usually*			
Inside home	209 (96.3)	8 (3.7)	0.592
Outside home	158 (95.2)	8 (4.8)	

*Lived in Metema*			
Less than 6 months	23 (95.8)	1 (4.2)	0.999
More than 6 months	345 (95.8)	15 (4.2)	

*History of travel*			
Yes	170 (94.4)	10 (5.6)	0.201
No	198 (97.0)	6 (3.0)	

*Seasonal migrant laborers*			
Yes	152 (96.2)	6 (3.8)	0.762
No	216 (95.6)	10 (4.4)	
